# Mast cell density and its clinical relevance in Waldenström's macroglobulinemia

**DOI:** 10.1002/jha2.378

**Published:** 2022-02-08

**Authors:** Richard Lemal, Stéphanie Poulain, Albane Ledoux‐Pilon, Lauren Veronese, Andrei Tchirkov, Benjamin Lebecque, Thomas Tassin, Jacques‐Olivier Bay, Frédéric Charlotte, Florence Nguyen‐Khac, Marc Berger, Catherine Godfraind, Loïc Ysebaert, Frédéric Davi, Bruno Pereira, Véronique Leblond, Olivier Hermine, Romain Guièze, Franck Pagès, Olivier Tournilhac

**Affiliations:** ^1^ Laboratoire d'Histocompatibilité, Centre de Biologie, CHU de Clermont‐Ferrand Université Clermont Auvergne Clermont Ferrand France; ^2^ Hématologie Clinique et Thérapie Cellulaire CHU Clermont‐Ferrand EA7453 CHELTER CIC1405 Université Clermont Auvergne Clermont Ferrand France; ^3^ “CANcer Heterogeneity Plasticity and Resistance to THERapies” INSERM 1277‐CNRS 9020 UMRS 12 University of Lille Lille France; ^4^ Service d'Hématologie Cellulaire Centre de Biologie Pathologie Lille France; ^5^ Anatomie Pathologique CHU Clermont‐Ferrand Université Clermont Auvergne Clermont Ferrand France; ^6^ Service de Cytogénétique Médicale CHU Clermont‐Ferrand INSERM U1240 IMOST Université Clermont Auvergne Clermont Ferrand France; ^7^ Service d'Hématologie Biologique CHU Clermont‐Ferrand Université Clermont Auvergne Clermont Ferrand France; ^8^ Anatomie Pathologique APHP La Pitié Salpêtrière Paris France; ^9^ Service d‘Hématologie Biologique Sorbonne Université Hôpital Pitié‐Salpêtrière Centre de Recherche des Cordeliers Paris France; ^10^ IUCT Oncopole Hématologie Clinique Toulouse France; ^11^ La Pitié Salpêtrière APHP Laboratoire d'Hématologie Paris France; ^12^ Direction de la recherche clinique Unité Biostatistique Clermont Ferrand France; ^13^ Hématologie Clinique APHP UPMC La Pitié Salpêtrière Paris France; ^14^ Hématologie Clinique APHP, IMAGINE Institute Necker‐Enfants Malades Paris France; ^15^ Immunomonitoring Plateform APHP Hôpital Européen Georges Pompidou Paris France

**Keywords:** mast cells, tumor biology, Waldenström's macroglobulinemia

## Abstract

The presence of numerous mast cells (MCs) mixed with tumor cells in the bone marrow (BM) is a hallmark of the diagnosis of Waldenström's macroglobulinemia (WM). MCs have been shown to support lymphoplasmacytic cell growth, but there is thus far no demonstration of the prognostic impact of BM MC density in WM. We investigated BM MC density by sensitive and specific digital quantification, allowing the analysis of a large area infiltrated by BM tumor cells. A total of 65 WM patients were investigated, including 54 at diagnosis and 11 at relapse. Tryptase and CD20 immunohistochemisty staining was performed on contiguous sections of deparaffinized BM trephine biopsies. After numerization of each section, the BM surface area was manually marked out, excluding the bone framework and adipocytes to limit the analyses to only hematopoietic tissue. MCs were assessed using a digital tool previously used to quantify immune‐cell infiltrates on tumor‐tissue sections. Deep next‐generation sequencing and allele‐specific PCR were used to explore the *MYD88* and *CXCR4* mutational status. MC density was heterogeneous among the WM patients. An optimal MC density threshold (> 56 MC.mm^–2^) was defined according to ROC curve analysis of overall survival. A higher MC density (> 56 MC.mm^–2^) was associated with greater BM involvement by WM lymphoplasmacytic cells and less hepatosplenic involvement (*p* = 0.023). Furthermore, MC density significantly correlated with a higher ISSWM score (*p* = 0.0003) in symptomatic patients. Patients with a higher MC density showed shorter median OS (56.5 months vs. nonreached, *p* = 0.0004), even in multivariate analysis after controlling for other predictive variables, such as age, ISSWM score, and *CXCR4* mutational status. In conclusion, MC density can be accurately measured in WM patients using a specific digital tool on well‐outlined hematopoietic tissue surfaces. High MC density is associated with aggressive features and a poor clinical outcome, emphasizing the need for further investigation of the involvement of MCs in the pathophysiology of WM.

## 1 INTRODUCTION

Waldenström's macroglobulinemia (WM) is characterized by lymphoplasmacytic infiltration of the bone marrow (BM), along with the presence of a serum monoclonal IgM. *MYD88* L265P and *CXCR4* mutations have been reported in > 90% and ≈ 25% of WM cases, respectively [1, 2, 3, 4, 5, 6]. The *MYD88* L265P mutation results in the constitutive activation of NFκB by Bruton's tyrosine kinase and is thought to be a clonal driver mutation [1]. The *CXCR4* mutations are mostly nonsense or frameshift mutations that occur most often in the S338 position. They result in truncation of the cytoplasmic portion of the receptor for the chemokine CXCL12, similarly to *CXCR4* mutations seen in WHIM (warts, hypogammaglobulinemia, infections, and myelokathexis) syndrome. Such truncation leads to impaired internalization of the receptor after ligation and results in prolounged activation [5]. *CXCR4* ^WHIM^ mutations are subclonal and associated with greater BM involvement, more symptomatic disease, and higher genomic complexity [6, 7, 8].

One historical hallmark for WM histological diagnosis is the presence of numerous mast cells (MCs) mixed with the tumor cells in the BM [9]. MCs are myeloid‐derived cells that are widely disseminated throughout all tissues and act as sentinels of the surrounding environment. They store and can release a wide spectrum of biologically active mediators after activation, leading to their central role in allergic diseases. The presence of numerous MCs in human tumors is well described and their pleiotropic molecule production is thought to explain their pro‐oncogenic roles (recently reviewed in [10, 11]). In hematological malignancies, MCs have been shown to be associated with progression and poor (or even good) prognoses in various Hodgkin's and non‐Hodgkin's lymphomas [12, 13, 14, 15, 16, 17, 18, 19]. In WM, MCs have been shown to support lymphoplasmacytic cell growth through CD154/CD40 signaling [20]. MC density in WM has already been explored by optical microscopy, showing a median of 49 MC.mm^–2^ (0.4–149.5), in contrast to a median of 14 MC.mm^–2^ (3.8–31.4) found in heatlhy subjects [21]. In this series, MC density was shown to significantly increase after treatment for nonresponders, to remain stable for minor responders, and to significantly decrease for major responders.

There has thus far been no clear demonstration of the clinical relevance nor prognostic impact of BM MC density in WM. We thus investigated BM MC density by sensitive and specific digital quantification, allowing the analysis of a large area of the BM infiltrated by tumor cells, to assess its clinical relevance in WM.

## 2 MATERIALS AND METHODS

### 2.1 Patients and samples

Sixty‐five patients with available BM trephine biopsy for WM at the Clermont‐Ferrand and La Pitié‐Salpêtrière University Hospitals were included between 1998 and 2012 in accordance with the Declaration of Helsinki. All included patients presented with WM as defined by the international guidelines [2, 3, 4], that is, BM infiltrated by an immunoglobulin M‐producing clonal lymphoplasmacytic lymphoma. Fifty‐four of the included patients had never been treated before the BM trephine biopsies, whereas 11 had already received treatment for WM. The median follow‐up from BM trephine biopsy was 69 months (1–133 months). Fifty‐three of 65 patients became symptomatic during the follow up after the BM trephine biopsy and were treated according to the Athens criteria [4]. Thirty of the 53 symptomatic patients (56.6%) received chlorambucil and/or rituximab monotherapy; 23/53 (43.4%) received chemoimmunotherapies with a backbone of cyclophosphamide and fludarabine or bendamustine. The patient characteristics are shown in Table 1A.

**TABLE 1:** Patient characteristics (A) general population and (B) according to MC density

	All patients	Low MC density	High MC density	*p*‐value
No. of patients	65	27	38	
Treatment naive, *N* (%)	54 (84)	26 (96)	28 (74)	**0.02**
Previously treated, *N* (%)	11 (16)	1 (4)	10 (26)	
Sex ratio (% male)	63	52	71	
Age at diagnosis, mean [range], years	60 [36–81]	57.5 [36–79]	65.4 [52–81]	**0.0026**
ISSWM (*n* = 43)				
low risk, *N* (%)	18 (42)	10 (67)	3 (10)	**0.0007**
intermediate risk, *N* (%)	13 (30)	3 (20)	15 (54)	
high risk, *N* (%)	12 (28)	2 (13)	10 (36)	
Clinical presentation				
Adenopathy, *N* (%)	24 (37)	11 (41)	13 (34)	0.59
Hepatosplenomegaly, *N* (%)	15 (23)	9 (33)	6 (16)	**0.023**
Hyperviscosity, *N* (%)	10 (15)	4 (15)	6 (16)	1
Neuropathy, *N* (%)	9 (14)	5 (19)	4 (11)	0.472
Fever, *N* (%) (*n* = 51)	3 (6)	1 (6)	2 (6)	1
Weight loss, *N* (%) (*n* = 49)	13 (27)	3 (18)	10 (31)	0.498
Cryoglobulinemia, *N* (%) (*n* = 39)	14 (36)	6 (35)	8 (36)	0.667
Mutational status				
*MYD88 L265P, N (%)*	49/55 (89.1)	17/21 (81)	32/34 (94.1)	0.19
*CXCR4 MUT, N (%)*	15/53 (28.3)	6/19 (31.6)	9/34 (26.5)	0.76
Biology				
Hb level ≤ 11,5 g/dl, *N* (%)	39 (60)	12 (44)	27 (71)	**0.031**
Hb, mean [range], g/dl	10.8 [5.4–15.2]	11.7 [6.3–15.2]	10.2 [5.4–15]	**0.02**
Platelet count < 100 X 109/L, *N* (%)	6 (9)	0	6 (16)	**0.037**
Platelet count, mean [range], X 109/L	224 [18–439]	247 [115–439]	208 [18–422]	0.11
β2 microglobulin > 3 mg/L, *N* (%) (*n* = 50)	25 (50)	9 (41)	16 (57)	0.254
β2 microglobulin, mean [range], mg/L (*n* = 50)	3.33 [1.4–9.8]	3 [1.4–6.3]	3.6 [1.7–9.8]	0.12
Monoclonal component > 70 g/L, *N* (%)	2 (3)	0	2 (5)	0.507
Monoclonal component, mean [range], g/L	28.4 [2.3–68.9]	28.2 [7–66.4]	28.5 [2.3–68.9]	0.95
Histopathological characteristics				
Lymphoplasmocytic infiltration > 60%	22 (34)	5 (19)	17 (45)	**0.0352**
Diffuse tumor pattern	18 (28)	3 (11)	15 (40)	**0.0132**

^*^bold values are for statistically significant data

### 2.2 Evaluation of MC density

Immunostaining was centrally performed at the Clermont‐Ferrand pathology laboratory using a monoclonal mouse anti‐human CD20 antibody (clone L26; Dako, Carpinteria, CA, USA, 1/100 dilution for 1 h) to stain WM tumor cells and a monoclonal mouse anti‐human tryptase antibody (clone AA1; Dako, 1/1000 dilution for 24 min) to stain MCs. Anti‐CD20 and antitryptase immunostaining was performed on two contiguous sections of BM trephine biopsies to assess the tumor infiltration characteristics and compare them to the MC infiltration pattern.

**FIGURE 1:**
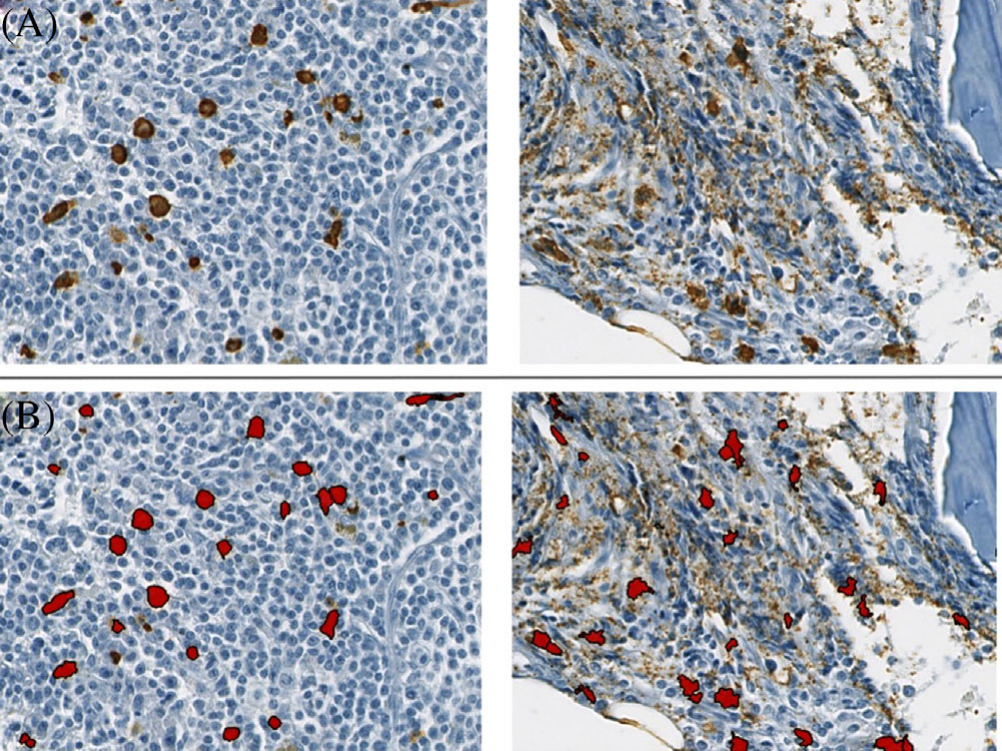
Sensitive and specific detection of tryptase‐positive cells with the immunoscore module. (A) BM detected with the anti‐tryptase Ab. Cells marked in brown are MCs. The background signal is shown in the right panel. (B) MC detection with the immunoscore module. Cells marked in red are the detected MCs. The background signal (not detected as MCs) is shown in the right panel

All computer‐based analyses were conducted using the “Immunoscore module” from Definiens Developer XT, which has already been used to quantify immunological infiltrates within tumors [22]. After digitization, each antitryptase immunostained section was manually marked out. Bone framework, adipocytes, and technical artifacts were excluded to limit the analyses to only hematopoietic tissue. We then defined the optimal thresholds for horseradish peroxidase signal detection to increase sensitivity while retaining specificity (Figure 1). The MC density was then automatically recorded as the number of tryptase‐positive cells per mm^2^ of the selected BM tissue surface area. For each BM section, we systematically reviewed random zones to verify whether the detection threshold was appropriate and avoid background noise.

For four patients, MC density was evaluated on two to four consecutive available BM trephine biopsies using the same protocol.

### 2.3 Molecular analyses for *MYD88* and *CXCR4* mutational status

Tumor DNA was available for 55/65 patients (84.6%), including 45/54 untreated (83.3%) and 10/11 previously treated (90.9%) patients. Fifty‐three samples were assessed by deep next‐generation sequencing for *MYD88* and *CXCR4* mutations, as previously described [23], allowing better assessment of the percentage of allele variants with 1% sensitivity. Two samples were assessed by allele‐specific PCR for the *MYD88* L265P mutation only.

### 2.4 Statistical analyses

The therapeutic requirements and time to reach the endpoints were defined according to published recommendations [4, 24, 25]. Statistical analyses were performed using SPSS Statistics v22 (IBM), PRISM v8.0 (Graphpad), and/or R software [26]. The Pearson Chi‐square, two‐sided Fisher, Mann–Whitney, and Kaplan–Meier tests with Log‐rank and Cox multivariate models were applied to the data as appropriate. MC density was first treated as a quantitative parameter. Then, a sensitivity analysis was conducted to categorize MC density according to its statistical distribution (i.e., median and interquartile range) and then ROC curve analysis, applying Youden's index, to determine the optimal threshold based on overall survival (OS).

## 3 RESULTS

### 3.1 The distribution of MC density and its correlation with deep tumoral infiltration

**FIGURE 2:**
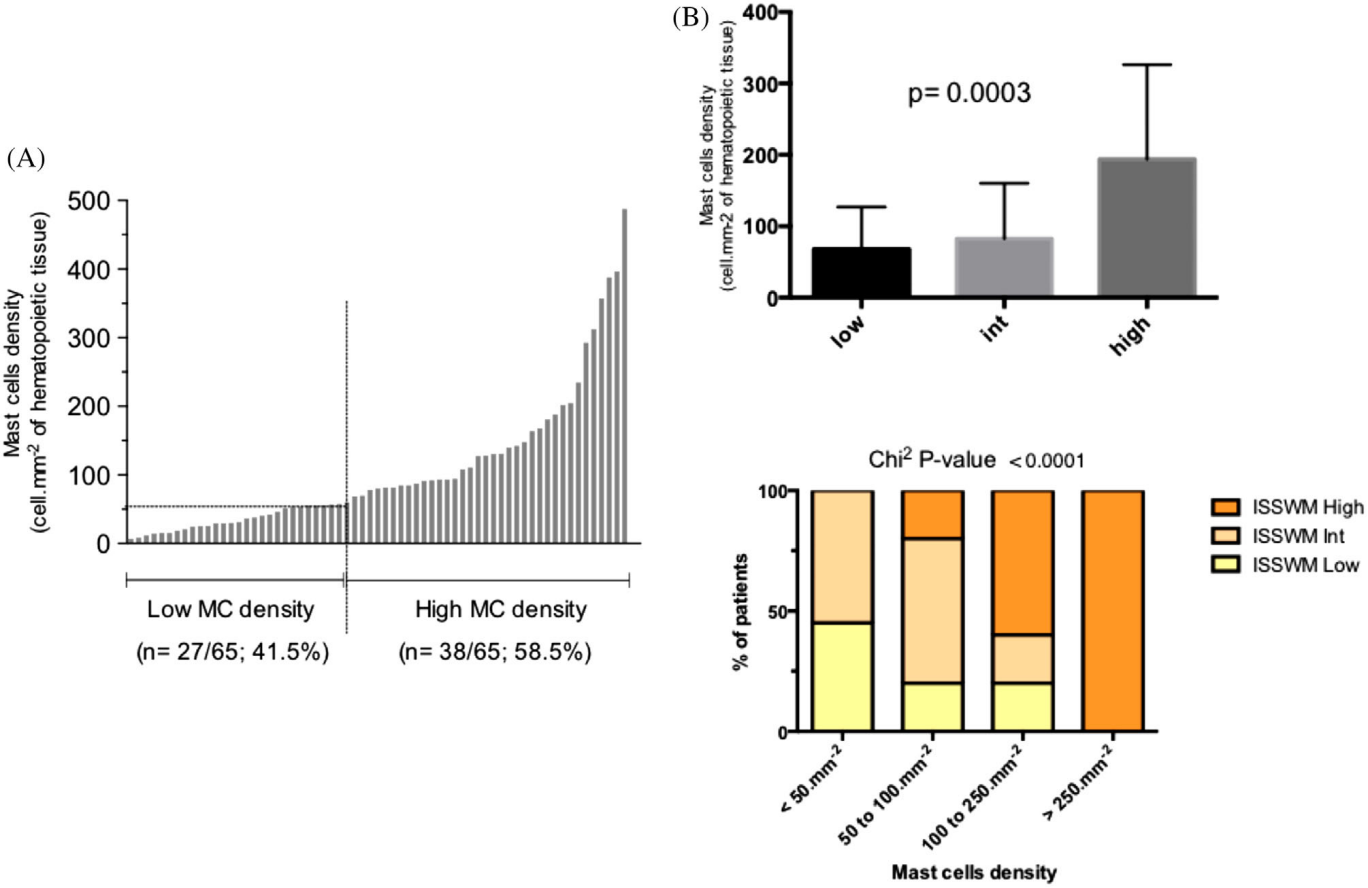
Mast cell density in WM patients. (A) Distribution of MC density in 65 WM (optimal MC density threshold = 56 MC.mm^–2^). (B) MC density correlates with the ISSWM score

The MC density ranged from 6.7 to 487 MC.mm^–2^ (mean 106.1 MC.mm^–2^, median 79.9 MC.mm^–2^). The optimal threshold was defined to be 56 MC.mm^–2^ by ROC curve analysis based on OS, pinpointing two populations with a high *versus* low MC density (Figure 2A).

A high MC density correlated with BM tumor involvement (*i.e*., lymphoplasmocytic infiltration > 60% of the BM surface) (*p* = 0.0352) and a diffuse tumor pattern (*p* = 0.0132).

### 3.2 The association of MC density with clinical and biological features

The clinical and biological features of the patients are shown in Table 1B according to their MC density. There was a statistical association between MC density and the ISSWM prognosis score (*p* < 0.001) (Figure 2B). Patients with a high MC density had significantly less hepatosplenic involvement (*p* = 0.023) but higher anemia (< 115 g.L^–1^; *p* = 0.031) and more thrombocytopenia (< 100 g.L^–1^; *p* = 0.037). High MC density was also statistically associated with an older age at diagnosis (65.4 years [52–81] *vs*. 57.5 years [39–69]; *p* = 0.0026) and previous treatment for the disease (*p* = 0.02).

**TABLE 2:** Details of CXCR4 mutations in this series

Nucleotide change	Amino acid change	Variant allele frequency
c.1000C > T	Arg334Ter	41
c.976dupC + c.1013C > A	Leu326Pro + Ser338Ter	1.5 + 12
c.1013C > A	S338X	2
c.1013C > G	S338X	45
c.1013C > A	S338X	7
c.1013C > G	S338X	25
c.1013C > A	S338X	1
c.1013C > G	S338X	3
c.1017dupT	V340C	2
c.1025_1026delCT	T342R	34
c.1013C > A	S338X	24
c.1021delT	S341P	12
c.1013delC	S338Y	31
c.1013C > G	S338X	4
c.968_969delGG	G323V	31

### 3.3 The association of MC density with the *MYD88* nor *CXCR4* mutational status

The *MYD88* L265P mutation was detected in 49 of the 55 tested patients (89.1%), including 39/45 (86.7%) who were treatment naïve and 10/10 (100%) who had been previously treated. Fifteen of the 53 tested patients (28.3%) carried a WHIM‐like mutation in *CXCR4*, including 13/43 (30.2%) who were treatment naïve and 2/10 (20%) who had been previously treated (details of the *CXCR4* mutations are shown in Table 2). All *CXCR4* mutations were observed in MYD88L265P‐mutated WM. MC density did not correlate with *MYD88* nor *CXCR4* mutational status but *MYD88* WT patients showed a trend toward a lower MC density (40.9 *vs*. 90.6 for *MYD88* L265P patients, *p* = 0.0742; size effect 0.61 [–0.24; 1.45]).

### 3.4 Impact of MC density on survival

**FIGURE 3:**
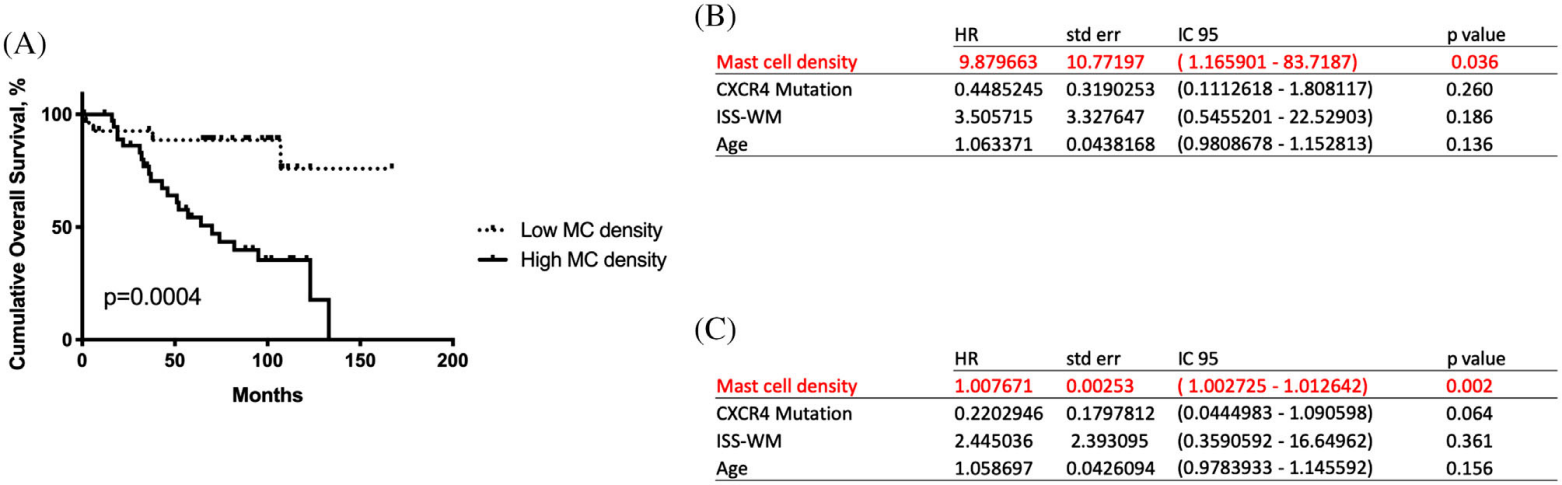
MC density affects overall survival in univariate and multivariate analyses. (A) Univariate analysis. (B) Multivariate analysis (MC density as a categorial variable). (C) Multivariate analysis (MC density as a continuous variable)

High MC density was associated with a poor clinical outcome, shown by shorter OS (56.5 months *vs*. not reached in patients with low MC density; *p* = 0.0004) (Figure 3A). OS was still significantly shorter for patients with high MC density in multivariate analysis after controlling for other predictive variables, such as age and ISSWM score in first‐line symptomatic patients and *CXCR4* mutational status, with MC density as a categorical (with a 56 MC.mm^–2^ cutoff) (Figure 3B) or continuous variable (Figure 3C). MC density had no impact on time to treatment initiation, strength of response, progression‐free survival, nor time to next treatment.

### 3.5 Evaluation of MC density at successive timepoints

**FIGURE 4:**
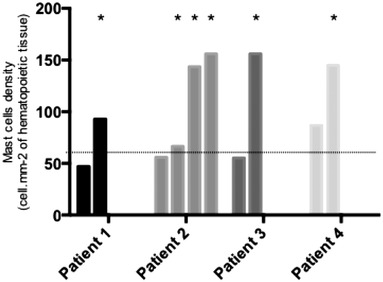
Sequential BM trephine biopsies of four patients. (*biopsies at subsequent relapse)

We evaluated MC density at various timepoints for four patients by examining one to four BM trephine biopsies during the clinical course of the disease (Figure 4). MC density increased upon relapse for all four patients.

## 4 DISCUSSION

Interactions between lymphoplasmacytic cells and the microenvironment, including MCs, are known to support their survival and proliferation. This study is the first to evaluate MC density using a sensitive and specific semi‐automated tool on large fields of well‐outlined hematopoietic tissue of the BM of Waldenström patients.

The distribution of MC density varied widely among patients. BM MC density in various hematological malignancies has been previously optically studied [21, 27, 28, 29]. Our results, based on this higher performing approach, are in line with the median of 49 MC.mm^–2^ (0.4–149.5) previously reported for 19 WM patients [21]. Some of our WM patients showed a very high MC density (up to 487.mm^–2^); such a high MC density has already been reported in cases of myelodysplastic syndrome using using optical methods (median of 214 MC.mm^–2^ [50–507.mm^–2^]) [30], illustrating the reliability of our method. Interestingly, while previously MC density evaluations have been optically studied with a focus toward tumor infiltrates and its periphery, we choose to evaluate MC density at all hematopoïetic areas with this digital solution, in order to deliver a more impartial and accurate anaysis. Namely, this allows us to accurately evaluate MC density for patients with intertitial infiltrate (even with paratrabecular aggregates) –that is, with lymphoid infiltrate being, by itself, uneasy to define.

We provide statistical evidence that MC density is associated with greater BM tumor involvement (i.e., infiltration > 60% of the BM surface) (*p* = 0.0352) and a diffuse tumor pattern (*p* = 0.0132), in accordance with the litterature on the pathological description of WM [9, 21, 30, 31], emphasizing the place of MCs as a diagnostic tool of the disease. Neither of these two histological criteria were associated with the (1) clinical presentation, (2) biological aggressivity, nor (3) outcome of the WM patients. Thus, MC density appears to be the only histopathological criterion to have such prognostic value.

MC density was statistically associated with an older age at the diagnosis of WM, relapse status, and a higher ISSWM score. In addition, we evaluated MC density at various timepoints for a selected group of four patients during the clinical course of the disease: MC density appeared to increase upon subsequent relapse, suggesting that MCs accumulate during WM and may be involved in disease relapse. MC density should be sequentially evaluated during the clinical course of WM (before and after treatment and at subsequent relapses) to clearly assess the impact of treatment(s) on MCs and/or the involvement of MCs in relapses.

MC density was associated with several features of more aggressive disease, such as anemia and thrombocytopenia, which are consistent with an association between high MC density and greater tumor involvement in the BM. These data, and the previously described bilateral communication between WM tumor cells and MC [20], suggest that (1) MCs may help to segregate lymphoplasmacytic cells in BM and/or (2) the WM tumor cells are directly responsible for the increase in MC density. These bilateral interactions are yet to be explored.

MC density was a pejorative prognostic factor for OS in our series. We evaluated age, relapsing disease, ISSWM score, and *CXCR4* mutational status by multivariate analysis, as these criteria can clearly affect OS. MC density was still the only criterion that affected OS, when considering it as a categorial or continuous variable after controlling for age, ISSWM score, and CXCR4 mutational status in first‐line patients.

The proportion of patients carrying *MYD88* and *CXCR4* mutations in our series was as expected [1, 5]. MC density was not associated with *MYD88* nor *CXCR4* mutational status in our series but the number of patients may have been insufficient to show such an association. Interestingly, WT *MYD88* patients showed a trend toward a lower MC density, arguing for a possibly different involvement of MCs in the pathophysiology of WT *MYD88* WM. This, however, has yet to be evaluated in an independent series.

All treated patients in our series received classical therapy with alkylating agents or chemoimmunotherapy. Currently, targeted therapies are increasingly used in this setting, namely, Bruton's tyrosine kinase inhibitors, such as ibrutinib, and the BCL2 inhibitor venetoclax, which is in clinical development. The clinical relevance of MC density and its evolution under such therapy still needs to be explored.

MC density can be accurately measured in WM patients using a specific digital tool on a well‐outlined hematopoietic tissue surface. High MC density is associated with several aggressive features, BM tumor burden, and poor clinical outcome. Further studies are necessary to precisely characterize the crosstalk between MCs and tumor cells in WM and evaluate the impact of WM therapies on MCs.

## AUTHOR CONTRIBUTIONS

RL, SP, AL, AT, BL, TT, FP, and OT performed the research. RL, SP, YL, VL, BP, OH, RG, FP, and OT designed the research study. FP, AT, JOB, FC, FNGK, MB, and CG contributed to essential reagents or tools. RL, SP, RG, OH, FP, and OT analyzed the data. RL, SP, and OT wrote the paper.

## CONFLICTS OF INTEREST

The authors have no conflicts of interest to declare.
